# Mago nashi controls auxin‐mediated embryo patterning in Arabidopsis by regulating transcript abundance

**DOI:** 10.1111/nph.70154

**Published:** 2025-04-18

**Authors:** Liping Liu, Wen Gong, Regina Stöckl, Philipp Denninger, Uwe Schwartz, Mark A. Johnson, Thomas Dresselhaus

**Affiliations:** ^1^ Cell Biology and Plant Biochemistry, Institute of Plant Sciences University of Regensburg Regensburg D‐93053 Germany; ^2^ Plant Systems Biology, School of Life Sciences Technical University of Munich Emil‐Ramann‐Strasse 8 Freising 85354 Germany; ^3^ NGS Analysis Center, Biology and Pre‐ClinicalMedicine University of Regensburg Regensburg D‐93053 Germany; ^4^ Department of Molecular Biology, Cell Biology, and Biochemistry Brown University Providence RI 02912 USA

**Keywords:** Arabidopsis, auxin, EJC, embryogenesis, Mago nashi, signaling, splicing

## Disclaimer

The New Phytologist Foundation remains neutral with regard to jurisdictional claims in maps and in any institutional affiliations.

Establishing the apical‐basal body axis is one of the earliest steps in embryo development of animals and plants. In the fruit fly *Drosophila*, for example, the axis is established by localized graded determinants in an initial syncytium (Bloom, 1996; Moussian & Roth, 2005). In vertebrates, axis formation is established through a sequence of interactions between neighboring cells and via cell movement (Czirok *et al*., 2004; Mongera *et al*., 2019). By contrast, plant embryogenesis has no syncytial phase, and each cell has a fixed position and does not move (Capron *et al*., 2009). In the model plant *Arabidopsis thaliana* (Arabidopsis), body axis formation is already initiated with the asymmetric division of the zygote. Zygotic division gives rise to a smaller apical daughter cell from which most of the embryo will develop and a large basal daughter cell, which will form the suspensor connecting the embryo to the maternal tissue. Two principal pathways regulate the establishment of the apical‐basal axis in Arabidopsis: one involves activation of the transcription factors *WUSCHEL‐RELATED HOMEOBOX 2* (*WOX2*) and *WOX8*, and the other one involves PIN‐FORMED (PIN)‐mediated auxin transport and temporal activity of the auxin response machinery (Lau *et al*., 2012; Robert *et al*., 2013; Palovaara *et al*., 2016; Dresselhaus & Jürgens, 2021). *WOX2* and *WOX8* are initially co‐expressed in the zygote and are thereafter restricted to the apical and basal daughter cells, marking the apical and basal cell lineages, respectively (Haecker *et al*., 2004; Breuninger *et al*., 2008). Plants utilize directional transport of auxin to generate an asymmetric auxin response that specifies the embryonic apical‐basal axis (Friml *et al*., 2003; Weijers *et al*., 2006; Ueda *et al*., 2011). Suspensor‐expressed auxin efflux carrier PIN7 mediates polar auxin flow from the suspensor toward the embryo proper, which is required for embryo development (Friml *et al*., 2003; Robert *et al*., 2013). Later during embryogenesis, the onset of localized auxin biosynthesis mediates polarization of the auxin efflux carrier PIN1, which is required for the specification of basal embryonic structures (e.g. the root pole) (Ikeda *et al*., 2009; Eklund *et al*., 2010; Robert *et al*., 2013).

Mago nashi (Mago), which was originally identified in *Drosophila*, is required for polarity establishment during early embryogenesis. Mago is a maternal component of an mRNP complex that is required for polarized localization of *oskar* mRNA to the posterior pole for axis formation in *Drosophila* oocytes (Boswell *et al*., 1991; Newmark & Boswell, 1994; Micklem *et al*., 1997). In mammalian cells, it was later shown that human Mago (MAGOH) together with RNA‐binding protein Y14, RNA‐binding protein metastatic lymph node 51 (MLN51), and eukaryotic initiation factor 4A‐III (eIF4AIII) form the core of the exon junction complex (EJC), a complex of more than 11 proteins that is deposited 20–25 nucleotides upstream of exon–exon junctions on spliced mRNAs (Zhang & Krainer, 2007). In addition to splicing, the EJC regulates nonsense‐mediated mRNA decay (NMD), nuclear mRNA export, and translation in mammalian cells (Mitra *et al*., 2023). Misexpression of MAGOH is correlated with the progression of certain cancers as it is required to safeguard the splicing of cell division and cell cycle genes; thus, MAGOH is now considered an oncogene (Barreiro *et al*., 2023; Yu *et al*., 2024).

Plant homologs of Mago show > 80% similarity in protein sequence with their animal counterparts and are encoded in most plant species by a single‐copy gene (Gong *et al*., 2014b; Ihsan *et al*., 2015). Plant *Magos* show a ubiquitous expression pattern in all tissues (Swidzinski *et al*., 2001; He *et al*., 2007; Ihsan *et al*., 2015). Like their mammalian EJC counterparts, Arabidopsis Mago (*At*Mago) forms a protein complex with *At*Y14 and Partner of Y14‐Mago (*At*PYM) in the nucleus (Pendle *et al*., 2005; Park & Muench, 2007) and was recently identified in the RNA‐binding proteome of an egg‐cell‐like callus (Liu *et al*., 2023). Arabidopsis Mago plays a role in spermatogenesis in early land plants (He *et al*., 2007; van der Weele *et al*., 2007; Boothby & Wolniak, 2011) and was shown to be required in flowering plants for male gametophytic functions and seed development, as shown for rice and Arabidopsis, respectively (Johnson *et al*., 2004; Park *et al*., 2009; Gong *et al*., 2014a; Cilano *et al*., 2016). However, the function of *At*Mago in early embryo development and polarity establishment, as well as the role of the EJC in posttranscriptional regulation of gene expression during embryogenesis, remains unclear.

In this study, we report early embryo patterning defects in a knockout mutant of *At*Mago named *hapless1* (*hap1‐1*) (Johnson *et al*., 2004) in Arabidopsis. We demonstrate that the defect in apical‐basal axis formation in *hap1‐1* is associated with the misexpression of *WOX8* and altered auxin response maximum. Additionally, RNA‐seq analysis of isolated *hap1‐1* globular embryos revealed significant alterations in the level of transcripts for proteins involved in auxin signaling, cell division, and RNA‐processing pathways. Moreover, genes associated with embryo development displayed differential alternative splicing (AS) patterns in *hap1‐1*, potentially contributing to the observed embryonic defects.

**Fig. 1:**
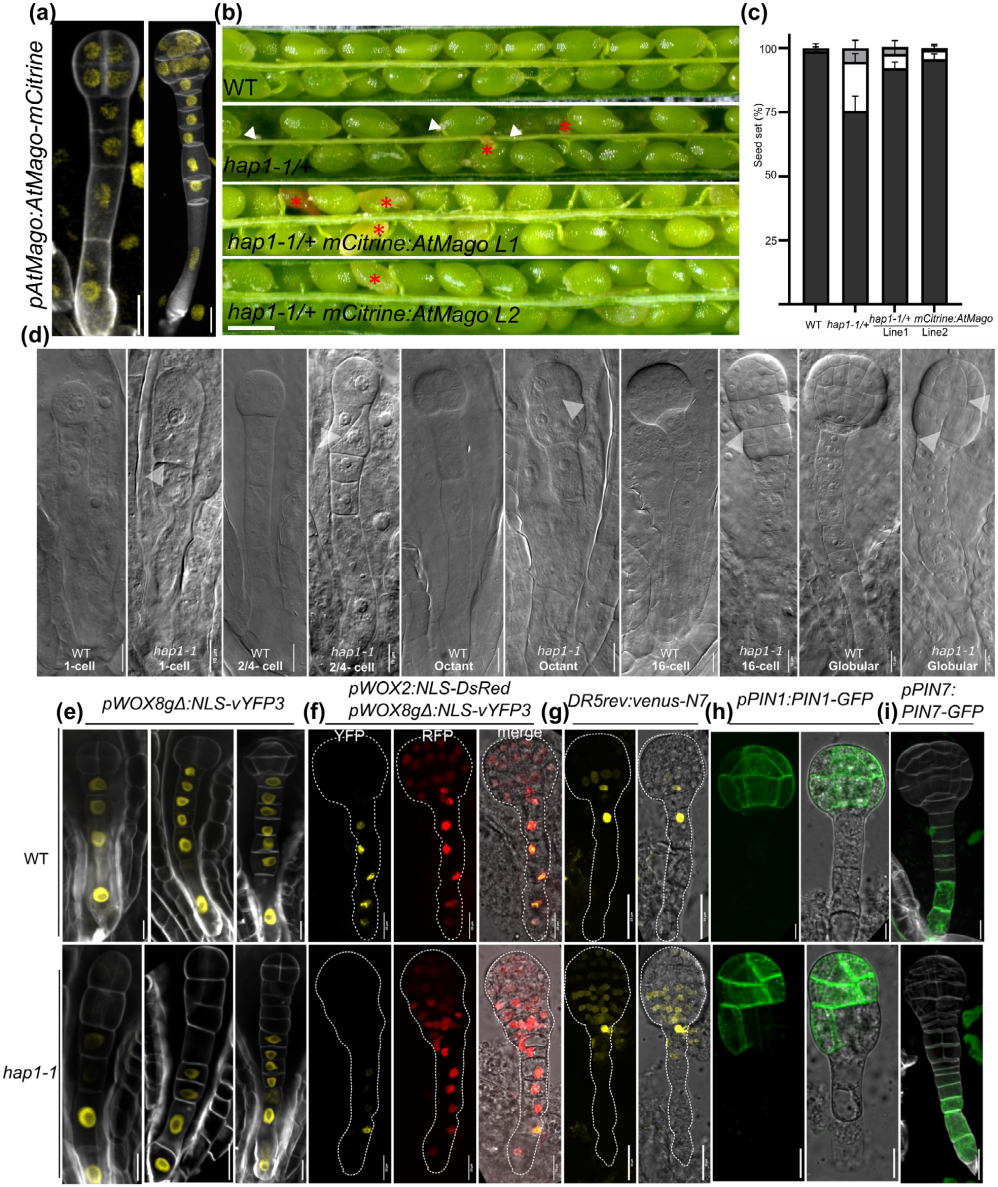
Embryo patterning is defective in Arabidopsis Mago *(AtMago)* (*hapless1 (hap1‐1)*) mutants of Arabidopsis. (a) Expression pattern and nuclear localization of *pAtMago:AtMago‐mCitrine* during early embryo development. Octant and dermatogen stages are shown. (b) Dissected siliques of wild‐type (WT), *hap1‐1*/+, and two *hap1‐1*/+ *mCitrine‐AtMago* lines. The white arrowheads indicate unfertilized ovules, and the red asterisks mark undeveloped seeds. (c) Seed set quantification for WT, *hap1‐1*/+, and two *hap1‐1*/+ *mCitrine‐AtMago* lines. Dark gray indicates developed seeds, white shows partially developed seeds, and light gray indicates unfertilized ovules. (d) Morphology of embryos at indicated stages in WT and *hap1‐1* mutants. White arrowheads indicate abnormal cell division of embryos. (e) Expression pattern of *pWOX8gΔ:NLS‐vYFP3* in WT (upper) and *hap1‐1* (lower) embryos. (f) Expression pattern of *pWOX2:NLS‐DsRed2/ pWOX8gΔ:NLS‐vYFP3* in globular stage embryos of WT (upper) and *hap1‐1* (lower) plants. (g–i) Expression pattern of *DR5rev:venus‐N7*, *pPIN1:PIN1‐GFP*, and *pPIN7:PIN7‐GFP* in WT and *hap1‐1* embryos. Bars: (a, d, e–i) 10 μm; (b) 50 μm.

To analyze the expression pattern of *At*Mago (*AT1G02140*), we first generated a *pAtMago:Atmago‐mCitrine* reporter line by using 2.5k‐bp upstream of the start codon as a promoter fragment. Arabidopsis Mago is expressed in all cells throughout female gametogenesis from the megaspore mother cell to the mature female gametophyte stage and localizes to the nucleoplasm. After fertilization, *AtMago* is ubiquitously expressed from the 2‐ to 4‐cell stage toward the late globular stage of the embryo (Fig. 1a, Supporting Information Fig. S1a,b). To address whether *AtMago* is required for embryo development, we used the T‐DNA insertion mutant *hap1‐1/+*, which is known to be homozygous lethal (Johnson *et al*., 2004). In the segregating progeny of *hap1‐1/+*, 76.3% of ovules developed into wild‐type (WT)‐like seeds, 18.5% into shrunken seeds, whereas 5.2% (*n* = 286) remained unfertilized (Fig. 1b,c). In the segregating progeny of *hap1‐1/+*, about one‐quarter (24.6%, *n* = 203) of embryos displayed more symmetric cell division at the 1‐cell stage (Fig. 1d). At the 2‐ to 4‐cell stage, about one‐quarter (25.4%, *n* = 216) of embryos displayed an oblique division pattern in the hypophysis. Subsequently, a proembryo‐like structure was formed at the presumptive hypophysis position (19.4%, *n* = 67) (Fig. 1d). Embryo defects of *hap1‐1* were largely complemented by *pAtMago‐mCitrine:AtMago* (still 7.69% defects, *n* = 117) (Fig. 1b,c). This observation indicates that the N‐terminal fusion of the reporter slightly disturbs *At*Mago function, which may be caused due to disturbance of the larger fusion protein to form a highly efficient EJC. This hypothesis is supported by the finding that a C‐terminal fusion protein was not capable of complementing the mutant (data not shown). Altogether, these findings indicate that AtMago is required for embryo development, especially for early embryo patterning.

WOX2 and WOX8 were previously reported to regulate the apical and basal cell lineage, respectively (Ueda *et al*., 2011). In consideration of the early embryo patterning defects of *hap1‐1*, we hypothesized that the expression pattern of *WOX8* would be affected in *hap1‐1*. To test this hypothesis, we crossed a *pWOX2:NLS‐DsRed, pWOX8gΔ:NLS‐vYFP3* double reporter into *hap1‐1/+*. In segregating progeny of *hap1‐1/+*, about one‐quarter (23.2%, *n* = 43) of embryos showed reduced *WOX8* expression in the descendant cells of the hypophysis with an abnormal cell division pattern (18.9%, *n* = 37), suggesting an altered cell fate in these cells (Fig. 1e,f). *pWOX2:NLS‐DsRed* expression pattern in *hap1‐1* was comparable to that in WT embryos (Fig. 1f).

Next, we addressed whether auxin response and transport are affected in *hap1‐1*, since auxin signaling and transport play critical roles during early embryogenesis (Friml *et al*., 2003). At the globular embryo stage, the strongest auxin response visualized by *DR5rev* activity was detected in the lens‐shaped cell, the hypophysis, and the uppermost suspensor cell in WT embryos (Fig. 1g, upper panels). However, in the progeny of *hap1‐1*, auxin maxima were expanded toward the descendant cells of the hypophysis, which correlates with an irregular cell division pattern at this region (17.3%, *n* = 23) (Fig. 1g, lower panels). To examine whether auxin transport was affected in *hap1‐1*, we analyzed the expression and protein localization of two major auxin efflux carriers: PIN1 and PIN7. Compared with a symmetrical and evenly distributed PIN1‐GFP localization in WT globular stage embryos, PIN1‐GFP localized unevenly and asymmetrically in *hap1‐1* mutants correlating with embryo defects (18.2%, *n* = 44) (Fig. 1h). By contrast, PIN7‐GFP expression in the lower suspensor cells remained unchanged (Fig. 1i). These findings suggest that *At*Mago is required for apical‐basal axis formation and patterning during early embryogenesis via maintaining the expression of *WOX8* in the basal cell lineage, including the hypophysis. Moreover, *At*Mago is required for correct PIN1 localization and auxin patterning at the globular stage.

**Fig. 2:**
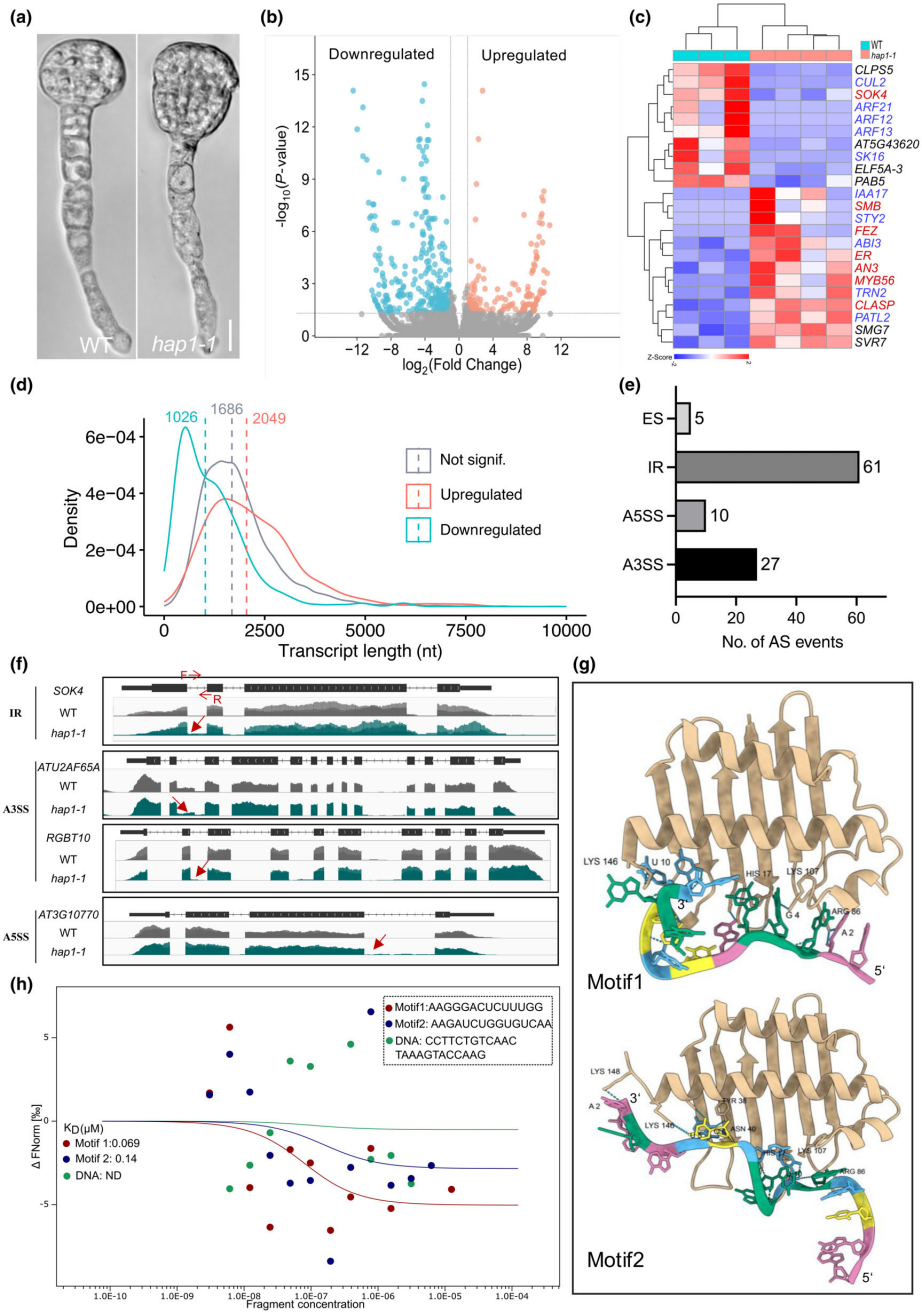
RNA‐seq analysis of early embryos display only minor splicing effects in Arabidopsis *hapless1 (hap1‐1)* mutant. (a) Isolated early globular embryos from wild‐type (WT) and *hap1‐1* plants. (b) Volcano plot of differently expressed genes (DEGs) in *hap1‐1*. (c) Heatmap plot of genes enriched in auxin response (text in blue), cell division (text in red), and RNA‐processing (text in black) in three WT and four *hap1‐1* samples. High relative transcript levels are indicated in red and low levels in blue. (d) Kernel density plot showing transcript length distribution of down‐ (cyan)/upregulated (red), or not significantly (gray) DEGs in *hap1‐1* compared with WT embryos. Dashed lines indicate the median of each group. (e) Alternative splicing (AS)‐type distribution of significantly different AS events in *hap1‐1*. (f) Selected genes containing significantly different AS events in *hap1‐1* (visualized via IGV viewer). Abnormal splicing regions are indicated by arrows. (g) Prediction of Arabidopsis Mago (*At*Mago) and RNA fragments interaction via Alphafold3. Predicted structures of *At*Mago bound to RNA reveal a concave binding pocket enriched in positively charged residues (Lys107, Lys146, His17, and Arg86) that interact with the RNA phosphate backbone. Arabidopsis Mago is shown in ochre. 5′ and 3′ ends of RNAs are indicated, and ‘A’, ‘U’, ‘C’, and ‘G’ bases are shown in pink, light blue, yellow, and green, respectively. (h) Arabidopsis Mago and RNA fragment interaction and the DNA control were measured by microscale thermophoresis. Datapoints indicate the difference in normalized fluorescence (%) generated by indicated RNA fragments and random DNA with fluorescently labeled *At*Mago. Curves show calculated fits. Curves in green, red, and blue represent DNA, RNA Motif1, and RNA Motif2, respectively. A3SS, alternative 3′ splice site; A5SS, alternative 5′ splice site; ES, exon skipping; IR, intron retention. Bar, 50 μm.

To elucidate the molecular mechanisms underlying *At*Mago‐associated posttranscriptional regulation of gene expression and to identify the downstream target genes/transcripts, we isolated defective globular stage embryos from *hap1‐1/+* plants (presumptive *hap1‐1* homozygotes) together with isolated WT globular stage embryos and performed RNA‐seq (Fig. 2a). Four hundred and forty‐seven differentially expressed genes (DEGs) were found, of which 160 were upregulated and 287 were downregulated in *hap1‐1* compared with WT embryos (|Log_2_FoldChange| ≥ 1; *P*_adj_ < 0.05) (Fig. 2b; Table S1). Genes with significantly altered expression patterns include those involved in auxin responses, cell division, and RNA‐processing (Fig. 2c). Among the auxin response category, several auxin response factor (*ARF*) genes including *ARF13*, *ARF12*, and *ARF21* were downregulated, while the auxin/indole‐3‐acetic acid (Aux/IAA) family member *IAA17* was upregulated in *hap1‐1*. This might be correlated with the expanded auxin maxima observed in defective embryos. Additionally, there are several genes enriched in the ‘regulation of cell division’ Gene Ontology (GO) category. *ANGUSTIFOLIA 3* (also known as *GRF‐INTERACTING FACTOR 1*) previously reported to be involved in cell proliferation during leaf and flower development (Lee *et al*., 2009; Zhang *et al*., 2019) was upregulated, which might explain the additional cells observed in *hap1‐1* mutant embryos. The *SOSKEI* (SOK) family was reported to be crucial for polarity establishment during embryogenesis (Yoshida *et al*., 2019; van Dop *et al*., 2020). Of this family, only *SOK4* was downregulated in *hap1‐1*. Furthermore, microtubule‐associated protein CLASP, which is involved in cell division and cell expansion, was upregulated in *hap1‐1* (Fig. 2c). Additionally, we observed the upregulation of genes involved in RNA processing, including NMD decay factor *SMG7* (Fig. 2c), which is crucial for NMD. Disruption of *SMG7* causes embryonic lethality (Kerenyi *et al*., 2008; Lee *et al*., 2020), indicating a potential involvement of *At*Mago in the NMD pathway during early embryo development. Given the decreased expression pattern of *WOX8* in *hap1‐1* suspensor cells (Fig. 1e,f), its expression level was further confirmed as obviously reduced in current RNA‐seq data (Fig. S1c). Together, these findings suggest that *At*Mago is required for regulating gene expression of auxin signaling, cell division, and RNA‐processing pathways. The latter was further supported by the finding that the length of downregulated transcripts was significantly shorter than the average length of detected transcripts (Fig. 2d). This observation suggests that *At*Mago plays a role in RNA stabilization.

It was reported previously that presplicing induces stable modification of mRNP structures at the conserved site located 20–24 nucleotides upstream of exon–exon junctions in mRNA. This indirectly implicated Mago in splicing efficiency and mRNA quality control (Le Hir *et al*., 2000; Boothby & Wolniak, 2011; Oshizuki *et al*., 2022). Knockdown of EJC core proteins causes widespread AS changes in mammalian cells (Wang *et al*., 2014). Considering the conserved function of the EJC, we next explored whether AS is affected in *hap1‐1* embryos. A total of 6501 AS events were identified by using rMATS analysis (Shen *et al*., 2014). Of these, 3068 differential AS events (47.2%) were identified in *hap1‐1* embryos, encompassing 1609 gene loci (|IncLevelDifference| > 0.1) (Table S2). These splicing events were categorized into five types: alternative 5′ splice sites (A5SS), alternative 3′ splice sites (A3SS), intron retention (IR), mutually exclusive exons, and exon skipping (ES). Intron retention was the most prevalent category, constituting 50.5% of all events (Fig. S2a), consistent with previous reports that retained introns are a prominent feature of AS in Arabidopsis (Ner‐Gaon *et al*., 2004; Bai *et al*., 2015; Cui *et al*., 2017; Tu *et al*., 2022). Gene Ontology enrichment analysis of the genes with differential AS events showed categories including ‘DNA metabolic process’, ‘embryo development’, and ‘organophosphate metabolic process’ (Fig. S2b). In addition, 103 AS events, encompassing 92 gene loci, were significantly different in *hap1‐1* embryos (|IncLevelDifference| > 0.1; false discovery rate (FDR) < 0.05). These are 10 A5SS events, 27 A3SS events, 61 IR events, and 5 ES events (Fig. 2e; Table S3). Abnormal AS patterns in *hap1‐1* embryos were visualized for four genes: IR (*SOK4*), A3SS (*ATU2AF65A* and *RGBT1*), and A5SS (*AT3G10770*) (Fig. 2f). Furthermore, we examined the expression level of a retained intron from *SOK4* by using a primer pair located in a retained intron. We found that the level of the retained intron in *SOK4* was significantly increased in *hap1‐1* embryos, whereas the expression level in WT and complemented mutant lines were comparable (Fig. S2c). Given the prevalence of IR events, we next examined whether *At*Mago influences IR by altering U2‐mediated splice sites, which comprise the majority of splice sites in most eukaryotic genomes (Dietrich *et al*., 1997; Szczesniak *et al*., 2013). We generated a Seqlog plot from all detected IR and those altered in *hap1‐1*. We found that the 5′ (5′SS) and 3′ splice sites (3′SS) of the IR events in *hap1‐1* correspond roughly to the consensus, with enrichment of ‘GU’ and ‘AG’ motifs, respectively (Fig. S2d). These results indicated that the general splicing of U2‐type introns was not affected by a mutation in *At*Mago. As the EJC was reported to be deposited at a conserved position 20–24 nt upstream of splice junctions, we next examined the sequence from 19 to 30‐bp upstream of all detected IR events to assess any potential effects of *At*Mago on EJC deposition. Seqlogo plots revealed no obvious consensus sequences in the EJC deposition region (Fig. S2e), consistent with previous reports that EJC binding is sequence‐independent and mediated primarily by the DEAD box protein eIF4AIII (Shibuya *et al*., 2004; Singh *et al*., 2012). Importantly, eIF4AIII transcripts did not show altered expression in *hap1‐1*, suggesting that its binding preference may not be affected. To further investigate the role of *At*Mago in AS during embryo development, we focused on differential IR events, which represent the largest category of differential AS events. For these IR events, motif enrichment analyses were performed. The top two enriched motifs were subsequently selected for RNA interaction assays. First, modeling via AIphafold3 was performed to predict *At*Mago‐RNA interactions (Fig. 2g): According to the predictions, Motif1 (AAGGGACUCUUUGG) and Motif2 (AAGAUCUGGUGUCAA) are capable of binding through hydrophobic and electrostatic interactions, especially via positively charged residues (Lys107, Lys146, His17, and Arg86) that interact with the RNA phosphate backbone. Motif1 exhibits higher affinity due to superior geometric complementarity, a more optimized shape fit and interactions with all four positively charged residues. Coulombic stabilization and stacking interactions (e.g. A6 in Motif1) further enhance specificity, highlighting sequence‐dependent binding properties of *At*Mago (Figs 2g, S2f). To validate the prediction, microscale thermophoresis (MST) assays were performed confirming that *At*Mago exhibited a higher affinity for Motif1 (*K*_D_ = 0.069 μM) than for Motif2 (*K*_D_ = 0.14 μM), indicating preferential recognition. A control of random DNA (CCTTCTGTCAACTAAAGTACCAAG) did not show detectable binding (ND), confirming RNA and motif specificity of the interaction (Fig. 2h). These results indicate that while *At*Mago mutation does not globally disrupt splicing patterns in *hap1‐1* embryos, it likely plays a role in specific splicing of certain genes, such as genes containing ‘AAGGGACUCUUUGK’ and ‘AAGAUCUGGUGWCWA' motifs. Further research is required to confirm *At*Mago's involvement in AS to elucidate its precise mechanisms of action in embryo development.

In summary, we investigated the role of *At*Mago in Arabidopsis embryo development using the *hap1‐1/+* mutant. Our data revealed significant defects in embryo patterning correlated with reduced *WOX8* transcript levels in the basal cell lineage. Furthermore, the observed disrupted cell division patterns were consistent with altered expression of *PIN1* and *DR5* reporters in *hap1‐1* globular stage embryos. Transcriptomic analysis of isolated globular embryos defined specific genes whose abundance and/or splicing patterns require *At*Mago. Many of these genes are required for cell division and auxin signaling. Notably, eight genes known to regulate cell division were found to be unregulated in *hap1‐1*. These include the NAC‐domain protein genes *FEZ* and *SMB* that were shown to be required for columella stem cell divisions (Willemsen *et al*., 2008). While *FEZ* and *SMB* are upregulated in *hap1‐1* embryos (Fig. 2c), their potential contribution to the periclinal cell division defects and hypophysis expansion in *hap1‐1* remains speculative, as their functions in embryogenesis have not been studied extensively. In addition, *IAA17* was upregulated while three *ARFs* (*ARF12*, *ARF13*, and *ARF21*) were downregulated in *hap1‐1*. Auxin response factor*s*, as downstream mediators of the auxin response, either activate or repress target gene expression. Notably, *ARF13* has been implicated in hypophysis specification and suspensor cell identity (Rademacher *et al*., 2012), indicating that altered *ARF* expression or transcript stability in *hap1‐1* may cause the observed abnormal hypophysis specification. Further functional studies are required to determine the precise roles of altered *ARF*s and *IAA17* in embryo development. This study also identified 90 genes (*c*. 0.5% of all genes expressed in early embryos) showing significant AS events linked to the loss of *At*Mago function. This indicates that these AS events may contribute to the observed defects in *hap1‐1* embryo development, but overall the total number of mis‐spliced transcripts is relatively low. Notably, the length of downregulated transcripts was found to be significantly shorter in the mutant than the average length of all transcripts. This observation suggests a role of *At*Mago in RNA stability, although further confirmation is necessary to ascertain whether this preference is attributable to AS or transcript abundance. In conclusion, *At*Mago, a component of the EJC, appears to regulate the expression of genes required for apical‐basal specification within the Arabidopsis embryo predominantly by controlling transcript abundance rather than splicing.

## Materials and Methods

### Plant materials and growth conditions

*Arabidopsis thaliana* (L.) Heynh. (Arabidopsis) ecotype Columbia (Col‐3), mutants, and reporter lines were grown on soil under long‐day conditions (16 h light at 8500 lux, 21°C, and 65% humidity). The previously identified *hap1‐1/+* line (CS16307) (Johnson *et al*., 2004) is available in the Arabidopsis Biological Resource Center.

### Constructs for plant transformation

Primers used for cloning are listed in Table S4. For *pAtMago:AtMago‐mCitrine* and *pAtMago:mCitrine‐AtMago*, 2545‐bp upstream of the start codon of *At*Mago (*At1G02140*) was used as the *AtMago* promoter. The whole gene sequence without the stop codon was amplified from genomic DNA using primers listed in Table S4. Amplified fragments were cloned via the Greengate cloning system. Genomic sequences containing plasmids were assembled with other modules into the pGGZ003 vector (Lampropoulos *et al*., 2013). Transgenic plants were generated by applying the floral‐dip method (Clough & Bent, 1998). Three independent transgenic lines were each analyzed by confocal microscopy.

### Microscopy

Phenotypic analysis of WT and *hap1‐1/+* embryo development was made by using differential interference contrast microscopy (Zeiss, Jena, Germany, Imager.A2). Siliques at different developmental stages were dissected and further processed as previously described (Aida *et al*., 1997). Clearing of whole siliques (by using chloral‐hydrate : H_2_O : glycerol = 8 : 2 : 1 solution) was performed before observation.

The expression pattern of reporter lines *pWOX8gΔ:NLS‐vYFP*, *pPIN7:PIN7‐GFP* was observed after Clearsee treatment and SR2200 staining (Kurihara *et al*., 2021) using a spinning‐disk confocal laser microscope system (Visitron system VisiScope with CSU‐W1) equipped with an HC PL APO 40×/1.3 NA water objective. Note that the homeodomain sequence of *WOX8* was removed from the genomic sequence in the *pWOX8gΔ:NLS‐vYFP3* vector by a deletion of 425 bp including the first exon and the beginning of the second exon. At the 3′ end of the genomic sequence, a *Bgl*II site was introduced, replacing the STOP codon, and an NLS‐vYFP3 fragment was inserted. Expression of *pWOX2:NLS‐DsRed/pWOX8gΔ:NLS‐vYFP3*, *DR5rev:Venus*, and *pPIN1:PIN1‐GFP* in *hap1‐1* was observed directly after embryo dissection by using the above‐mentioned Visitron spinning‐disk system. *mCitrine* was excited at 505 nm, *DsRed2* was excited at 561 nm, SR2200 staining with DAPI was excited at 405 nm, and GFP was excited at 488 nm. *DR5rev* was used as a synthetic auxin‐responsive element to monitor auxin response maxima.

### Embryo isolation

Embryo isolation was carried out following a previously described method with slight modifications (Zhou *et al*., 2019). Self‐pollinated siliques were harvested for collecting ovules for embryo isolation. Siliques were dissected under the stereoscopic microscope, and ovules were transferred to 100 μl of enzyme solution in the bottom of a 3.5‐cm Petri dish. Ovules were treated with cell‐wall‐degrading enzyme solution for 30 min at room temperature as described (Zhou *et al*., 2019). Next, the enzyme solution was removed, and 100 μl of washing solution was added to wash ovules three times. Globular stage embryos were dissected directly from ovules with two fine needles under an inverted microscope (Eclipse TS100; Nikon, Düsseldorf, Germany). Isolated embryos were transferred to another droplet of washing solution by a handmade capillary pipette, then transferred to 10 μl of RNAlater (Thermo Fisher, Dreieich, Germany), and kept at −80°C for later usage. For segregating mutant embryos, only defective embryos were isolated. About 20 isolated embryos were each pooled as one replicate for RNA‐seq.

### Total RNA extraction and mRNAseq

Total RNA was extracted from isolated embryos and stabilized in RLT Plus buffer according to the protocol of the RNeasy Plus Micro Kit (Qiagen). In brief, cells were stored in 350 μl of buffer RLT Plus containing 1% ß‐mercaptoethanol and shipped on dry ice. After thawing, samples were homogenized by vertexing for 1 min. Genomic DNA contamination was removed by using gDNA Eliminator spin columns (Sigma‐Aldrich–MilliporeSigma). Next, one volume of 70% ethanol was added, and each sample was applied to RNeasy MinElute spin columns (Qiagen) followed by several washing steps. Finally, total RNA was eluted in 12 μl of nuclease‐free water. Purity and integrity of RNA were assessed using the Agilent 2100 Bioanalyzer with the RNA 6000 Pico LabChip reagent set (Agilent, Waldbronn, Germany).

Library preparation and mRNAseq were carried out as described in the SMART‐Seq mRNA LP User Manual (Takara Bio, Saint‐Germain‐en‐Laye, France), the Illumina NextSeq 2000 Sequencing System Guide (Illumina Inc., Cambridge, UK), and the KAPA Library Quantification Kit – Illumina/ABI Prism Protocol (Roche Sequencing Solutions Inc., Mannheim, Germany). In brief, *c*. 2 ng of total RNA was used to generate first‐strand cDNA. Double‐stranded cDNA was amplified by LD‐PCR (12 cycles) and purified via magnetic bead cleanup. After validating the quality and quantity, *c*. 150 pg of cDNA was enzymatically fragmented and stem‐loop adapters were ligated. Then, libraries were PCR‐amplified (16 cycles) and indexed, generating Illumina‐compatible libraries with unique dual indexes. After a magnetic bead purification, libraries were quantified using the KAPA Library Quantification Kit. Equimolar amounts of each library were sequenced on an Illumina NextSeq 2000 instrument controlled by the nextseq 2000 control software (NCS) v.1. 5.0.42699 using a 100 cycles P3 Flow Cell with the dual index, single‐read run parameters. Image analysis and base calling were made by the real time analysis software v.3.10.30 (Toh *et al*., 2017). The resulting .cbcl files were converted into .fastq files with the bcl2fastq v.2.20 software (www.illumina.com/company/legal.html).

### RNA‐seq analysis

Raw data of the fastq format were first processed through the fastp software (Chen *et al*., 2018). Clean data (clean reads) were mapped to the Arabidopsis TAIR10 genome using hisat2 v.2.0.5 (Kim *et al*., 2019). Transcripts of each sample were assembled by stringtie (v.1.3.3b) (Pertea *et al*., 2015) in a reference‐based approach. featurecounts v.1.5.0‐p3 (Liao *et al*., 2014) was used to count the read numbers mapped to each gene (GTF file). Reads per kilobase of exon model per million were quantified for gene expression level. Differential expression analysis of *hap1‐1* and WT was performed using the deseq2 R package (1.20.0) (Love *et al*., 2014). Resulting *P*‐values were adjusted using the FastLSU approach (https://github.com/cran/fastLSU) for controlling the FDR. Genes with an adjusted *P*‐value ≤ 0.05 and | log_2_FC | >1 found by DESeq2 were assigned as differentially expressed. Original code was applied by Novogene (https://www.novogene.cn/). shniygo 0.81 was used for GO enrichment analysis (http://bioinformatics.sdstate.edu/go/) FDR cutoff < 0.1.

The xstreme function of MEME suite (v.5.5.7) was used to identify enriched motifs in transcripts that showed significant IR effects (Table S3 ‘RI’) (Bailey *et al*., 2015). Transcript sequences of all expressed genes were used as background. The discovery of enriched motifs was restricted to 25 (‐‐streme‐nmotifs 25).

### Protein expression and purification

The coding sequence of *At*Mago was amplified and cloned into the pMAL‐c2X vector. A protocol of protein expression and purification from *Escherichia coli* (Liu *et al*., 2013) was modified as follows. In brief, *At*Mago‐MBP recombinant protein was expressed in *E. coli* strain BL21 (DE3). Protein expression was induced at 16°C, 180 rpm for 16 h with 0.2 mM isopropyl‐β‐D‐thiogalactopyranoside until the OD_600_ reached 0.4. Cells were harvested by centrifugation at 2400 ***g*** for 15 min, resuspended in column buffer (CB: 10 mM Tris–HCl pH 8.0, 150 mM NaCl, 1 mM EDTA, 1 mM PMSF, pH 8.0), and crushed using a low‐temperature ultrahigh‐pressure cell disrupter (JNBIO). Soluble proteins were separated from cell debris by high‐speed centrifugation (13 800 ***g*** for 1 h). Proteins were then loaded onto an amylose column (NEB, E8021S) and washed with 100 ml of washing buffer (CB). Protein was eluted from columns with CB containing 10 mM maltose.

### Microscale thermophoresis assay

Microscale thermophoresis (MST) assays were conducted according to previous studies (Yu *et al*., 2023). Purified *At*Mago protein (as discussed earlier) was labeled with RED‐NHS (Monolith™ RED‐NHS second‐generation protein labeling kit). RNA fragments (Motif1 and Motif2) and the random DNA fragment control were diluted into a range of concentrations from 10^−15^ to 10^−4^ M. Various concentrations of RNA or DNA solutions were mixed with 10 mM *At*Mago solution. The mixture solutions were incubated using an interaction buffer (100 mM NaCl, 1 mM EDTA, 20 mM sodium phosphate, pH 8.0). Samples were loaded into Monolith NT.115 Capillaries (MO‐K002; NanoTemper Technologies) using 50% IR laser power and an LED excitation source with λ = 470 nm at an ambient temperature. Dose–response curves for the interactions were calculated using the nanotemper analysis 1.2.20 software.

## Competing interests

None declared.

## Author contributions

TD and MAJ initiated the project. TD and LL designed the research, which was performed by LL, WG, RS and PD with bioinformatic support by US. LL and TD wrote the manuscript with input from all authors. All authors read and approved the final manuscript.

## Supporting information


**Fig. S1** Expression pattern of Arabidopsis Mago and *WUSCHEL‐RELATED HOMEOBOX 8* in Arabidopsis.
**Fig. S2** Alternative splicing events in the Arabidopsis Mago *hapless1* mutant at the early globular embryo stage of Arabidopsis.

**Table S1** Differently expressed genes in Arabidopsis *hapless1* embryos.

**Table S2** Differential alternative splicing events in Arabidopsis *hapless1* embryos.

**Table S3** Significantly differential alternative splicing events in Arabidopsis *hapless1* embryos.

**Table S4** Primer list.
Please note: Wiley is not responsible for the content or functionality of any Supporting Information supplied by the authors. Any queries (other than missing material) should be directed to the *New Phytologist* Central Office.

## Data Availability

All the data that support the findings of this study are available within the article and the Supporting Information (Figs S1, S2; Tables [Link], [Link], [Link], [Link]).
