# Supplementary material for: Mago nashi controls auxin‐mediated embryo patterning in Arabidopsis by regulating transcript abundance
Source: New Phytol. 2025 Apr 18;247(1):14–23. doi: 10.1111/nph.70154 (PMC12138180; doi:10.1111/nph.70154)
Supplement: Supplementary file 1 — Fig. S1 Expression pattern of Arabidopsis Mago and WUSCHEL‐RELATED HOMEOBOX 8 in Arabidopsis. Fig. S2 Alternative splicing events in the Arabidopsis Mago hapless1 mutant at the early globular embryo stage of Arabidopsis. [file NPH-247-14-s002.pdf]

New Phytologist Supporting Information

Article title: Mago Nashi controls auxin-mediated embryo patterning in Arabidopsis by regulating transcript abundance

Authors: Liping Liu, Wen Gong, Regina Stöckl, Philipp Denninger, Uwe Schwartz, Mark A. Johnson, Thomas Dresselhaus*

Article acceptance date: 22 March 2025

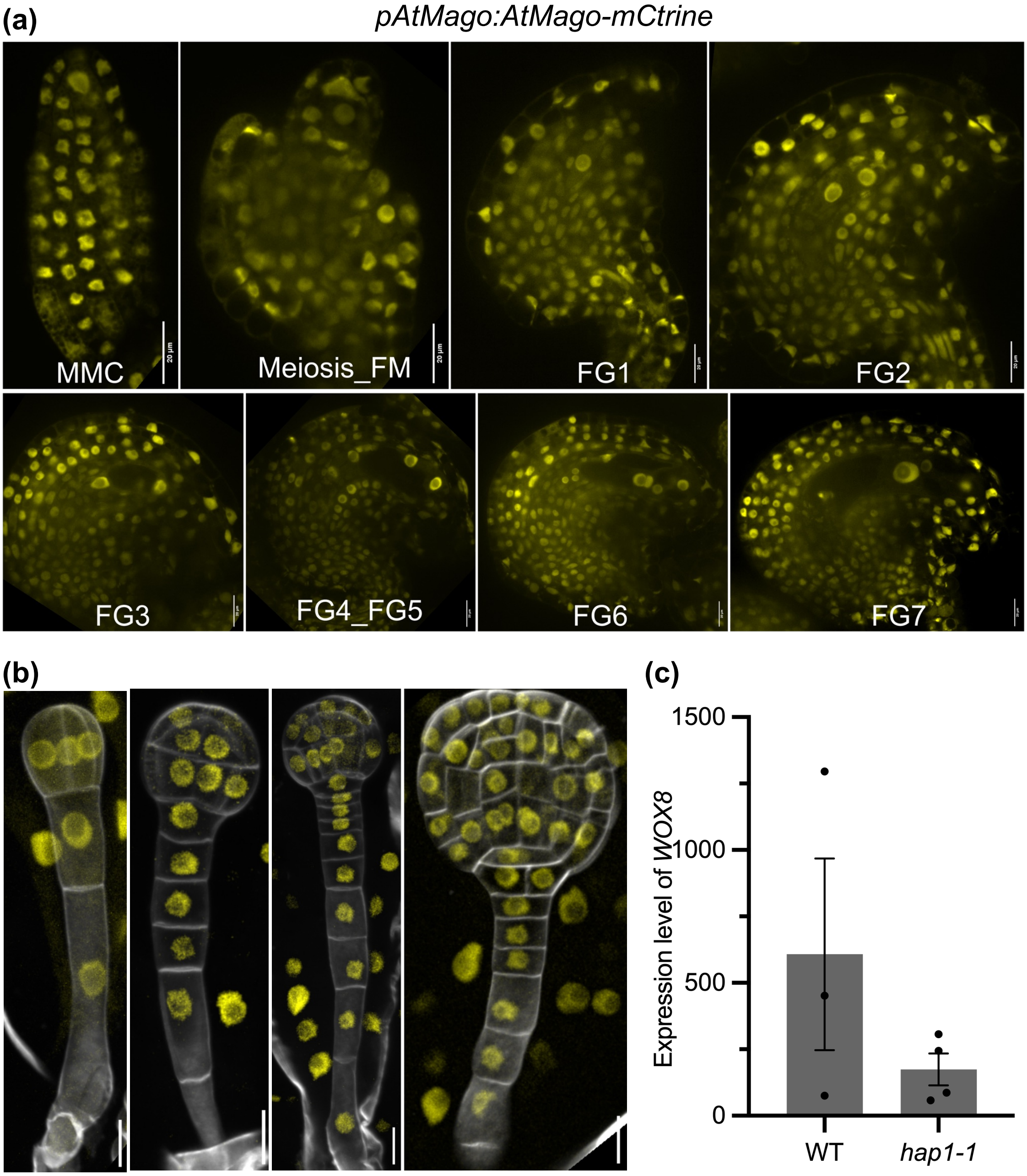

Fig. S1 Expression pattern of *AtMago* and *WOX8* in Arabidopsis. Expression pattern and subcellular localization of *pAtMago:AtMago-mCtrine* during ovule and female gametophyte (a) as well as during early embryo development (b). Embryos are shown at 4-cell, dermatogen, early and late globular stages, respectively. Abbreviations: FG, female gametophyte (number indicates stage); FM, functional megaspore; MMC, megaspore mother cell. Scale bars are 10 µm. (c) Expression level of *WOX8* in isolated embryos for WT and *hap1-1* mutant.

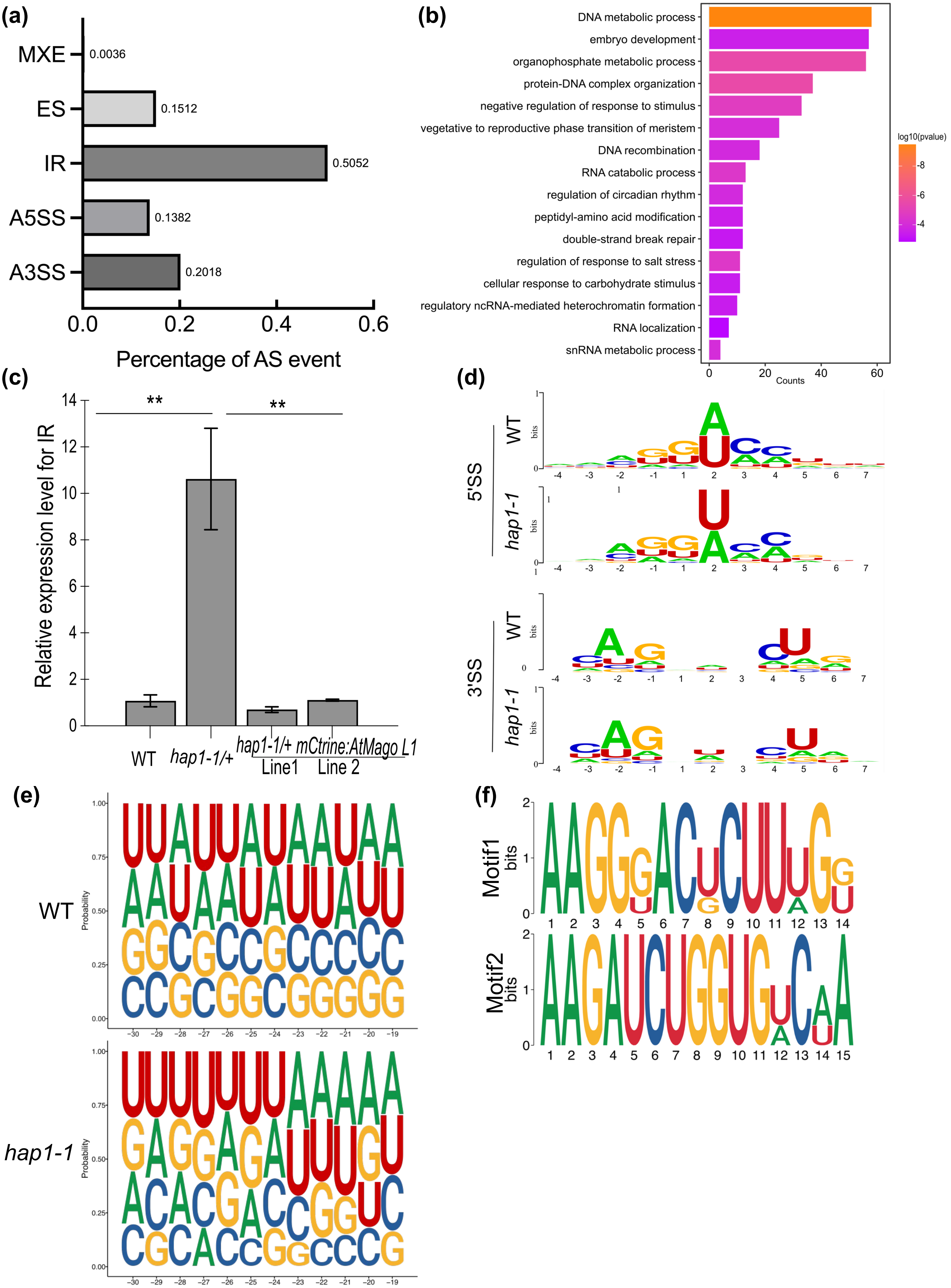

(a)
MXE
ES
IR
A5SS
A3SS
0.0036
0.1512
0.5052
0.1382
0.2018
0.0
0.2
0.4
0.6
Percentage of AS event
(b)
DNA metabolic process
embryo development
organophosphate metabolic process
protein-DNA complex organization
negative regulation of response to stimulus
vegetative to reproductive phase transition of meristem
DNA recombination
RNA catabolic process
regulation of circadian rhythm
peptidyl-amino acid modification
double-strand break repair
regulation of response to salt stress
cellular response to carbohydrate stimulus
regulatory ncRNA-mediated heterochromatin formation
RNA localization
snRNA metabolic process
0
20
40
60
Counts
log10(pvalue)
-8
-6
-4
(c)
Relative expression level for IR
14
12
10
8
6
4
2
0
**
**
WT
hap1-1/+
hap1-1/+ mCtrine:AtMago L1
Line1
Line 2
(d)
5'SS
WT
hap1-1
3'SS
WT
hap1-1
bits
-4
-3
-2
-1
1
2
3
4
5
6
7
(e)
WT
hap1-1
Probability
1.00
0.75
0.50
0.25
0.00
−30
−29
−28
−27
−26
−25
−24
−23
−22
−21
−20
−19
(f)
Motif1
Motif2
bits
2
1
0
1
2
3
4
5
6
7
8
9
10
11
12
13
14
15

Fig. S2 Alternative splicing (AS) events in the *At*Mago *hap1-1* mutant at the early globular embryo stage. (a) AS-type distribution of five different AS events. (b) GO enrichment analysis of transcripts containing different alternative splicing events in *hap1-1*. (c) Relative expression of retained intron of *SOK4* in different lines. Primer locus (F-R) is indicated in Figure 2f. Data are means ± SD (n = 3 biological replicates; **P < 0.01, Student's t-test). (d) 5` and 3` splicing sites of U2-type introns for IR events in WT and *hap1-1* embryos. (e) Seq-log plot at the EJC complex deposit region in WT and *hap1-1* embryos. (f) Motif enrichment analysis for different IR events in *hap1-1*. Abbreviations: A5SS, alternative 5′ splice site; A3SS, alternative 3′ splice site; ES, exon skipping; IR, intron retention; MXE, mutually exclusive exon.
